# Suicidal ideation during adolescence: The roles of aggregate genetic liability for suicide attempts and negative life events in the past year

**DOI:** 10.1111/jcpp.13653

**Published:** 2022-06-29

**Authors:** Séverine Lannoy, Becky Mars, Jon Heron, Alexis C. Edwards

**Affiliations:** ^1^ Department of Psychiatry, Virginia Institute for Psychiatric and Behavioral Genetics Virginia Commonwealth University School of Medicine Richmond VA USA; ^2^ Population Health Sciences University of Bristol Bristol UK

**Keywords:** ALSPAC, suicidality, genes, polygenic, environment

## Abstract

**Background:**

Suicidal thoughts and behaviors (STB) constitute a central public health concern in adolescence. Previous studies emphasized the difficulty to cope with negative life events during adolescence as a risk factor for STB. Familial and genetic liability has also been documented to explain STB risk. Nevertheless, less is known about aggregate genetic liability and its possible interaction with negative life events. Moreover, information is needed to understand how these factors differently affect STB in boys and girls.

**Methods:**

We evaluated suicidal ideation at 17 years old and examined the role of aggregate genetic liability, negative life events, and their interaction in a sample of 2,571 adolescents. Aggregate genetic liability was measured using a polygenic score (PGS) for suicide attempts. Negative life events were assessed in the past year and included parental divorce and hospitalizations, death of friends and relatives, bullying, failure‐related events, and involvement with drugs. We conducted univariable and multivariable general linear models stratified by sex and evaluated the interactions between PGS and negative life events in subsequent models.

**Results:**

Analyses showed that suicidal ideation in boys is associated with failure to achieve something important (estimate = 0.198), bullying (estimate = 0.285), drug use (estimate = 0.325), and parental death (estimate = 0.923). In girls, both aggregate genetic liability (estimate = 0.041) and negative life events (failure at school [estimate = 0.120], failure to achieve something important [estimate = 0.279], drug use [estimate = 0.395], and bullying [estimate = 0.472]) were associated with suicidal ideation. Interaction analyses suggested that PGS interacted with drug use and failures at school, though this would need additional support.

**Conclusions:**

These findings represent significant contributions to the fundamental understanding of STB in adolescence, suggesting to monitor the impact of negative life events during adolescence to better prevent suicide risk. Genetic liability is also of importance in girls and might influence the way they respond to environmental threats.

## Introduction

Suicidal thoughts and behaviors (STB) are highly prevalent during adolescence and encompass various forms. Suicidal thoughts and nonsuicidal self‐harm are predominant at this time (Gillies et al., 2018; Lawrence et al., 2021) and may lead to suicide attempt (Gillies et al., 2018; Mars et al., 2019a; Nock et al., 2009), whereas suicide is identified as a leading cause of death among adolescents (Shain, 2016; WHO, 2021). Prior research reported sex differences in STB, with suicidal thoughts and suicide attempts being more prevalent in girls and suicide deaths in boys (Fox, Millner, Mukerji, & Nock, 2018; Miranda‐Mendizabal et al., 2019; Simons & Murphy, 1985). In this study, we focused on suicidal ideation because it is highly prevalent in adolescence and constitutes a significant proximal predictor of suicide attempts (Nock et al., 2009). Offering a better understanding of the factors leading to suicidal ideation presents an opportunity to prevent suicidal behavior.

Adolescence is characterized by numerous changes, such as the development of identity, the ability to cope with stressful life events, and the initiation of risk‐taking. In the absence of fully developed coping skills, specific life events may prove especially challenging for young people and lead to STB (Rew, Young, Brown, & Rancour, 2016). Various life events have been related to STB in adolescence, namely, maltreatment, violence, loss, familial difficulties, as well as school and interpersonal problems (Serafini et al., 2015). Other proximal environmental factors have been documented, supporting the high risk associated with bullying (Holt et al., 2015; O'Reilly et al., 2021; Tang et al., 2020; Zaborskis, Ilionsky, Tesler, & Heinz, 2019), alcohol, and drug use (Mars et al., 2019a; Sher & Zalsman, 2005). Consistent with sex differences in stress reactivity (Henze et al., 2021; Liu & Zhang, 2020), prior evidence suggested that negative life events may differentially affect adolescent boys and girls (Coelho et al., 2018) and lead to different responses (Bordin, Handegard, Paula, Duarte, & Ronning, 2022; Tang et al., 2020). However, this needs further exploration in the context of STB.

A robust literature also shows that STB are prominently influenced by family history and genetic liability (Brent & Melhem, 2008; Shain, 2016). Familial resemblance is primarily influenced by genetic factors, with a modest role of shared environment (Edwards et al., 2021; Tidemalm et al., 2011). A meta‐analysis of twin studies indicated that the heritability estimates of suicide‐related phenotypes (suicidal thoughts, plans, and attempts) were between 30% and 55% (Voracek & Loibl, 2007), which has been supported by a recent study in a large population‐based cohort (Edwards et al., 2021). Among adolescents, the heritability of suicidal behaviors was estimated around 50% (Glowinski et al., 2001; Orri et al., 2020), with higher heritability in girls (Althoff et al., 2012). However, little is known regarding the possible interaction between this genetic risk and the way adolescent boys and girls react to negative life events. This lack of knowledge is due, in part, to the paucity of adequately powered genetic studies of STB, which can serve as ‘discovery samples’ for subsequent studies, until quite recently.

Several studies targeting functional candidate genes have suggested a role of gene–environment interactions in the etiology of STB (Brodsky, 2016; Roy, Sarchiopone, & Carli, 2009; Sanabrais‐Jimenez et al., 2019), but evidence in adolescence is inconsistent. Moreover, our conceptualization of psychopathology has evolved toward a polygenic nature, since (a) variation within candidate genes explains only a small proportion of the phenotypic variance and (b) replication of findings from candidate gene studies has been limited (Musci, Augustinavicius, & Volk, 2019). Recently, well‐powered studies have examined gene‐by‐environment genome‐wide interaction in adults (Wendt et al., 2021; Ye et al., 2021), which focus on individual genetic variants. Nevertheless, a dearth of information remains on how exposure to negative life events might exacerbate or attenuate the putative effect of genetic risk on the occurrence of STB in adolescence. Moreover, how this interaction plays out at the level of aggregate genetic liability, rather than at the level of individual small‐effect genetic loci, remains to be investigated. Here, we used the term aggregate genetic liability to refer to a polygenic score (PGS).

In this study, we evaluated the interaction between PGS for suicide attempts and the occurrence of negative life events between 16 and 17 years old on suicidal ideation. In view of sex differences in STB, possible reactions to negative life events, and genetic risks, we explored these effects in adolescent boys and girls separately. We used data from the Avon Longitudinal Study of Parents and Children (ALSPAC) and derived an index of suicidal ideation evaluating the presence of suicidal thoughts. Life events were evaluated in the past year with an inventory covering a wide range of familial, school, and interpersonal problems. As we were specifically interested in STB during adolescence, we computed a PGS based on a recent GWAS for suicide attempts (Mullins et al., 2021), which does not include nonsuicidal self‐harm thoughts and behaviors. We had three specific aims: (a) Evaluate the impact of aggregate genetic liability on risk for suicidal ideation in adolescent boys and girls; (b) Evaluate the impact of negative life events by targeting various environmental exposures on risk for suicidal ideation in boys and girls; (c) Determine whether genetic and environmental factors interact to predict suicidal ideation in adolescent boys and girls.

## Methods

We selected participants from the ALSPAC, a prospective observational study investigating development across the life course. This sample has been fully described previously (Boyd et al., 2013; Fraser et al., 2013) and the study website contains details of all the data that are available through a fully searchable data dictionary and variable search tool (http://www.bristol.ac.uk/alspac/researchers/our‐data/).

For the ALSPAC research, pregnant women resident in Avon (UK) with expected dates of delivery between 1st April 1991 and 31st December 1992 were invited to take part in the study. The initial number of pregnancies (Phase I) enrolled was 14,541, this included women who completed at least one questionnaire or attended a ‘Children in Focus’ clinic by 19/07/99. Among these pregnancies, there was a total of 14,676 fetuses, resulting in 14,062 live births and 13,988 children who were alive at 1 year of age. When the oldest children were approximately 7 years of age, an attempt was made to bolster the initial sample with eligible cases who had failed to join the study originally. As a result, when considering variables collected from the age of seven onwards (and potentially abstracted from obstetric notes) there are data available for more than the 14,541 pregnancies mentioned above. The number of new pregnancies was 913 (456, 262, and 195 recruited during Phases II, III, and IV, respectively), resulting in an additional 913 children being enrolled. The phases of enrolment are described in more detail in the cohort profile paper and its update (Boyd et al., 2013; Fraser et al., 2013). The total sample size for analyses using any data collected after the age of 7 is therefore 15,454 pregnancies and 15,589 fetuses (14,901 were alive at 1 year of age).

### Participants

In the current study, we focused on the clinical assessment youth completed at age 17 (TF4). A total of 10,085 adolescents were invited for this clinical assessment and 4,684 attended it. Around 2,571 participants were included in the final analyses (Figure S1). We used information on individuals invited to the TF4 assessment to correct for potential biases in the sample used for the current analyses (see Appendix S1).

Ethical approval for the study was obtained from the ALSPAC Ethics and Law Committee and the Local Research Ethics Committees. Informed consent for the use of data collected via questionnaires and clinics was obtained from participants following the recommendations of the ALSPAC Ethics and Law Committee at the time. Consent for biological samples has been collected in accordance with the Human Tissue Act (2004).

### Measures

We used suicidal ideation as an outcome variable and evaluated the predictive role of aggregate genetic liability (PGS) and negative life events that happened in the past year while controlling for socioeconomic status (SES). As prior genetic and phenotypic studies showed the role of SES in STB (Nock et al., 2009; Ye et al., 2021), we included maternal social class as an indicator of SES in our analyses. However, as ALSPAC is a community sample of youth, we did not incorporate additional control for psychopathology.

#### Suicidal ideation

This index has been computed based on the Computerized Interview Schedule – Revised (CIS‐R), assessing criteria for depression. This computerized interview has been validated to assess depressive symptoms and suicidal ideation in community samples (Lewis, Pelosi, Araya, & Dunn, 1992), but also among adolescents (Patton et al., 1999). The specificity and predictive validity of this instrument are very good and some studies used it to specifically assess suicidal ideation (Skapinakis et al., 2011; Stansfeld et al., 2017). During the interview, all adolescents (regardless of the level of depressive symptoms) were asked about passive suicidal thoughts and hopelessness in the past week. The evaluation offers a score ranging 0–3, indexing severity of suicidal ideation: 0, adolescents with no hopelessness or suicidal thoughts; 1, adolescents feeling hopeless but without suicidal thoughts; 2, adolescents feeling life is not worth living but without thoughts of death; and 3, adolescents with suicidal thoughts.

#### Life events

The life events inventory evaluates life events that happened to the adolescent in the past year with 38 questions covering familial events such as the move to a new neighborhood, the birth of a sibling, the loss of a job, the death of a pet, or parental divorce; school‐related events such as moving to a new school or having failed at school; health‐related events, such as hospitalizations or death of relatives, friends, or illness/injury of themselves; events related to having troubles with the police or being involved in accidents; events related to personal job; recognition events such as having reached excellence in an activity; affective‐related events such as break‐ups; conflict with the parents; pregnancy; bullying; failure‐related events, and drug‐related events. From this exhaustive list, we selected the negative events that have been related to STB in previous studies (Hua, Bugeja, & Maple, 2019; Mars et al., 2019b; O'Reilly et al., 2021; Serafini et al., 2015; Thompson, Alonzo, Hu, & Hasin, Thompson Jr., Alonzo, Hu, & Hasin, 2017), namely, parental divorce, hospitalizations or death of someone close, bullying, failure‐related events (failure at school and failure to achieve something important), and involvement with drugs.

#### Maternal social class

This variable was assessed during the pregnancy by asking the mother about her job occupation, status (i.e., foreman, manager, supervisor, leading hand, self‐employed, none of these), and services (i.e., main things done in job). The occupation was coded using the Office of Population Censuses and Surveys (OPCS; https://sru.soc.surrey.ac.uk/SRU9.html) job codes and the social class categorization was derived as an ordinal variable going 1–6 from lower to higher social classes, with a specific mention for job in armed forces.

### Polygenic liability

Genotypes are available for a subsample of ALSPAC participants (*n* = 8,729). ALSPAC analysts performed genotyping and initial quality control before the distribution of the data (not specific to the current study). ALSPAC children were genotyped using the Illumina HumanHap550 quad chip genotyping platforms by 23andMe subcontracting the Wellcome Trust Sanger Institute, Cambridge, UK and the Laboratory Corporation of America, Burlington, NC, US. The resulting raw genome‐wide data were subjected to standard quality control methods (see Appendix S1).

To derive PGS in the current study, we selected unrelated individuals from the ALSPAC sample. We used summary statistics from the most recent and well‐powered GWAS on suicide attempts (Mullins et al., 2021), from the International Suicide Genetics Consortium. PGS analyses were conducted using PRS‐CS, a method relying on a Bayesian regression framework and performing a continuous shrinkage prior on the effect sizes of SNPs (Ge, Chen, Ni, Feng, & Smoller, 2019). Continuous shrinkage was applied in the discovery GWAS summary statistics (Mullins et al., 2021) using 1,000 Genomes European as the LD reference panel with a Python‐based command line tool. Then, using the weighted summary statistics (*n* = 5,814,419 SNPs), we used PLINK 2.0 (Chang et al., 2015) to derive a PGS for each individual in our ALSPAC sample.

### Statistical analysis

To account for population stratification, we regressed PGS on the first 10 ancestral principal components. We then computed the standardized residuals from the regression model and used this corrected score in subsequent analyses. ALSPAC assessments are age‐standardized, therefore age was not accounted in the PGS computation nor included as a covariate. However, given prior literature on sex differences as well as recommendations from the National Institutes of Health to study sex‐specific effects (https://orwh.od.nih.gov/sex‐gender/nih‐policy‐sex‐biological‐variable), we explored how results varied according to biological sex. To support this approach, we first conducted analyses to evaluate interactions between sex and predictors (PGS and negative life events) in risk for suicidal ideation (see Appendix S2, Table S1).

To examine the association between PGS for suicide attempts and negative life events with suicidal ideation among adolescents, we first performed a series of univariable linear regressions stratified by sex. Then, we ran multivariable analyses including all variables (maternal social class, PGS, and negative life events) in the same model with a sex stratification. Finally, we investigated if PGS moderated the impact of negative life events in adolescents by using linear models. Interaction tests were only pursued where the main effects of the PGS and the life events had a *p* < .05 in the multivariable analyses. However, we followed previous recommendations advising to rely on estimates rather than *p*‐values (Amrhein, Greenland, & McShane, 2019) and described estimates for interactions with a *p* < .1. Analyses were performed with R 3.6.1, we used the ‘glm2’ package (https://cran.r‐project.org/web/packages/glm2/index.html) to fit our generalized linear models. As we relied on a large sample, we performed standard regressions with a Gaussian family distribution. We also performed ordinal logistic regressions with the ‘clm’ function (https://cran.r‐project.org/web/packages/ordinal/index.html) (see Appendix S2, Tables S2 and S3). Interaction plots were produced with the ‘effects’ package (https://cran.r‐project.org/web/packages/effects/index.html).

## Results

### Description of the sample

Table 1 reports descriptive characteristics of the sample, the mean PGS, and the prevalence of adolescents reporting each specific life event. Among the whole sample, 33.72% reported one negative life event, 18.13% reported two, 7.23% reported three negative life events, 1.36% reported four, and less than 1% of the sample reported 5 or 6 negative life events in the past year. The mean and SD for the index of suicidal ideation are also reported in Table 1.

**Table 1:** Description of the sample

Variables	Sample *n* = 2,571 *n* (%) or *M*(*SD*)	Boys *n* = 1,135 *n* (%) or *M*(*SD*)	Girls *n* = 1,436 *n* (%) or *M*(*SD*)	Difference between boys and girls (*t* or chi^2^)
Suicidal ideation	0.46 (0.73)	0.34 (0.66)	0.55 (0.77)	7.51***
Demographics				
SES (maternal social class)				12.97*
1	227 (8.83%)	112 (9.87%)	115 (8.00%)	
2	982 (38.19%)	445 (39.21%)	537 (37.39%)	
3	1,001 (38.93%)	442 (38.94%)	559 (38.93%)	
4	153 (5.95%)	64 (5.64%)	89 (6.20%)	
5	181 (7.04%)	61 (5.37%)	120 (8.36%)	
6	26 (<5%^a^)	10 (<5%^a^)	16 (<5%^a^)	
Armed forces	<5^a^	<5^a^	<5^a^	
Aggregate genetic liability				
PGS	−0.02 (1.02)	−0.03 (1.02)	−0.02 (1.02)	0.20^ns^
Life events in the past year				
Drug use	269 (10.46%)	151 (13.30%)	118 (8.22%)	16.97***
Bullying	119 (4.63%)	49 (4.32%)	70 (4.87%)	0.33^ns^
Parental death	8 (<5%^a^)	<5^a^	<5^a^	0.00^ns^
Grand‐parent death	330 (12.84%)	150 (13.22%)	180 (12.53%)	0.21^ns^
Friend death	163 (6.34%)	58 (5.11%)	105 (7.31%)	4.82*
Parent hospitalization	282 (10.97%)	134 (11.81%)	148 (10.31%)	1.31^ns^
Parental divorce	51 (<5%^a^)	23 (<5%^a^)	28 (<5%^a^)	0.00^ns^
Friend injury	223 (8.67%)	77 (6.78%)	146 (10.17%)	8.74**
Failure to achieve something important	745 (28.98%)	321 (28.28%)	424 (29.53%)	0.42^ns^
Failure at school	394 (15.32%)	176 (15.51%)	218 (15.18%)	0.03^ns^

^a^ Consistent with ALSPAC guidelines, we do not report the exact number for cells with count less than 5 (<5). This may include zero.

^ns^ = Not Significant, *p < .05; **, *p* < .01; ***, *p* < .001.

Preliminary analyses on the interaction between sex and PGS as well as sex and negative life events indicated that higher genetic liability was related to higher suicidal ideation in girls but not in boys (Figure S2), supporting the stratification of the analyses by sex. Interactions between sex, bullying, and possibly parental death were also found (Table S1).

### Univariable regression model

In boys, suicidal ideation was associated with SES, failure at school, parental hospitalization, failure to achieve something important, bullying, drug use, and parental death (estimates 0.021–1.023; Table 2). However, no association was found with aggregate genetic liability (PGS).

**Table 2:** Univariable regression models in boys (left; *n* = 1,135) and girls (right; *n* = 1,436) with suicidal ideation as outcome

	Boys				Girls			
	Estimate	95% CI	*t*	*p*‐Value	Estimate	95% CI	*t*	*p*‐value
SES	0.021	0.002; 0.039	2.28	.023	0.005	−0.032; 0.042	0.26	.793
PGS	−0.005	−0.043; 0.033	0.27	.787	0.057	0.017; 0.096	2.83	.004
Drug use	0.384	0.274; 0.495	6.81	<.001	0.473	0.332; 0.615	6.55	<.001
Bullying	0.362	0.176; 0.549	3.81	<.001	0.627	0.447; 0.808	6.82	<.001
Parental death	1.023	0.241; 1.803	2.57	.010	0.024	−0.657; 0.705	0.07	.945
Grand‐parent death	0.037	−0.074; 0.148	0.65	.514	0.047	−0.075; 0.168	0.75	.451
Friend death	0.079	−0.093; 0.252	0.90	.368	0.165	0.013; 0.318	2.12	.034
Parental hospitalization	0.132	0.014; 0.250	2.19	.029	0.122	−0.010; 0.254	1.81	.071
Parental divorce	0.110	−0.156; 0.376	0.81	.416	−0.022	−0.314; 0.269	0.15	.879
Friend injury	0.145	−0.006; 0.295	1.87	.061	0.152	0.018; 0.286	2.22	.026
Failure achievement	0.259	0.175; 0.343	6.04	<.001	0.343	0.257; 0.429	7.81	<.001
Failure at school	0.128	0.143; 0.287	2.38	.017	0.231	0.120; 0.342	4.09	<.001

The left part of the table shows univariable results in boys and the right part of the table indicates univariable results in girls.

In girls, PGS was associated with suicidal ideation (estimate = 0.057) as well as several negative life events (Table 2; estimates 0.152–0.627): friend injury, friend death, failure at school, failure to achieve something important, drug use, and bullying.

### Multivariable regression model

Among boys, suicidal ideation at age 17 was associated with specific negative life events in the past year, such as failure to achieve something important, bullying, drug use, or having experienced parental death (estimates 0.198–0.923). However, PGS was not associated with suicidal ideation in boys (Table 3).

**Table 3:** Multivariable regression models in boys (left; *n* = 1,135) and girls (right; *n* = 1,436) with suicidal ideation as outcome

	Boys				Girls			
	Estimate	95% CI	*t*	*p*‐value	Estimate	95% CI	*t*	*p*‐value
SES	0.017	−0.001; 0.035	1.95	.052	0.001	−0.034; 0.036	0.06	.953
PGS	−0.004	−0.041; 0.031	0.25	.802	0.041	0.004; 0.079	2.15	.032
Drug use	0.325	0.215; 0.435	5.80	<.001	0.395	0.255; 0.534	5.55	<.001
Bullying	0.285	0.103; 0.467	3.08	.002	0.472	0.293; 0.651	5.18	<.001
Parental death	0.923	0.166; 1.680	2.39	.017	0.086	−0.565; 0.738	0.26	.795
Grand‐parent death	0.035	−0.073; 0.143	0.63	.528	0.014	−0.102; 0.131	0.24	.809
Friend death	0.020	−0.148; 0.189	0.24	.810	0.091	−0.059; 0.256	1.18	.238
Parental hospitalization	0.101	−0.013; 0.216	1.74	.082	0.115	−0.013; 0.243	1.76	.079
Parental divorce	0.070	−0.187; 0.328	0.54	.592	−0.014	−0.293; 0.265	0.10	.919
Friend injury	0.094	0.054; 0.240	1.25	.212	0.003	−0.130; 0.136	0.05	.964
Failure achievement	0.198	0.113; 0.283	4.58	<.001	0.279	0.192; 0.366	6.27	<.001
Failure at school	0.065	0.039; 0.170	1.22	.223	0.120	0.010; 0.230	2.15	.032

The left part of the table shows multivariable results in boys and the right part of the table indicates multivariable results in girls.

In adolescent girls, suicidal ideation was associated with aggregate genetic liability (PGS; estimate = 0.041) and specific negative life events in the past year (Table 3), namely, failure at school, failure to achieve something important, drug use, and bullying (estimate = 0.120–0.472).

### Interaction between aggregate genetic liability and negative life events

**Figure 1:**
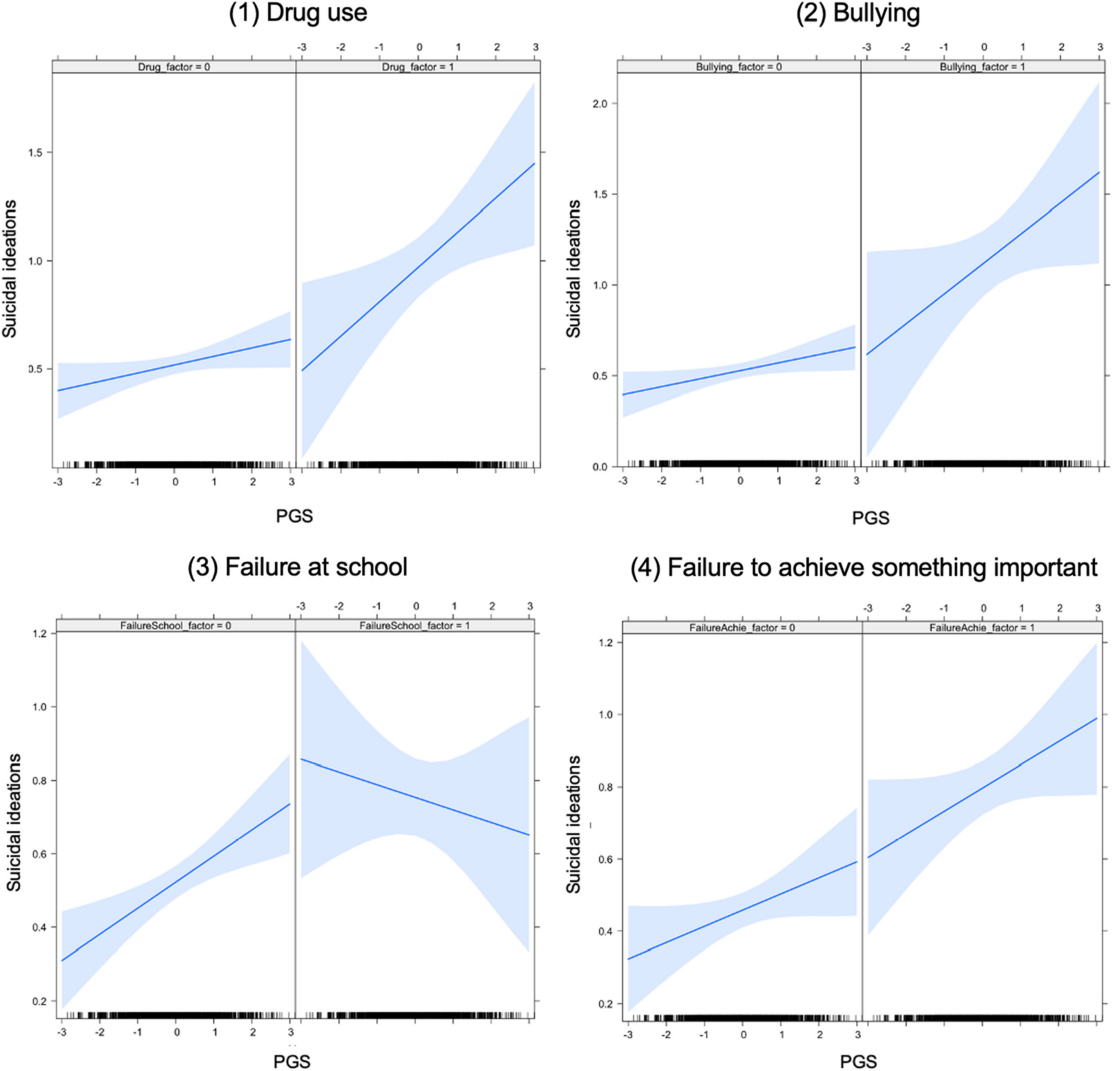
depicts the interaction between aggregate genetic liability (PGS) and negative life events (drug use, bullying, failure at school, failure to achieve something important) in adolescent girls. Negative life events are binary variables, with the absence of events on the left (0) and the presence of the event on the right (1). The solid blue line represents the interaction with PGS and the shaded area the 95% confidence intervals [Colour figure can be viewed at wileyonlinelibrary.com]

This set of analyses was restricted to girls given the absence of a main effect of PGS in boys, and used the negative life events that were associated with suicidal ideation in the multivariable model. Results are depicted in Figure 1 and suggested that aggregate genetic liability moderated the impact of drug use (estimate = 0.120) and failure at school (estimate = −0.105) in the past year. Specifically, drug use was more strongly associated with suicidal ideation among girls with high genetic liability to suicide attempts, and failure at school was more strongly associated with suicidal ideation among girls with low genetic liability to suicide attempts. Finally, no interactions were observed between PGS and bullying (estimate = 0.120) or the experience of failure to achieve something important (estimate = 0.020), see Table 4.

**Table 4:** Interaction between PGS and negative life events to predict suicidal ideation in girls (*n* = 1,436)

	Estimate	95% CI	*t*	*p*‐value
SES	0.0003	−0.036; 0.037	0.02	.987
PGS	0.039	−0.001; 0.080	1.90	.057
Drug use	0.452	0.310; 0.595	6.24	<.001
PGS × Drug use	0.120	−0.009; 0.249	1.83	.068
SES	−0.001	−0.037; 0.035	0.05	.963
PGS	0.043	0.004; 0.083	2.15	.032
Bullying	0.592	0.408; 0.775	6.32	<.001
PGS × Bullying	0.123	−0.049; 0.296	1.41	.160
SES	0.007	−0.029; 0.043	0.38	.705
PGS	0.044	−0.003; 0.091	1.85	.064
Failure achievement	0.339	0.253; 0.425	7.71	<.001
PGS × Failure	0.020	−0.062; 0.101	0.48	.633
SES	0.002	−0.034; 0.039	0.12	.907
PGS	0.071	0.029; 0.113	3.30	<.001
Failure at school	0.231	0.121; 0.342	4.10	<.001
PGS × Failure at school	−0.106	−0.215; 0.005	1.88	.061

## Discussion

Numerous studies have reported the role of negative life events (Howarth et al., 2020) and genetic liability (Tidemalm et al., 2011) in STB within adults but studies in adolescents are still somewhat limited. In this study, we evaluated the main effects of, and interactions between aggregate genetic liability for suicide attempts and the occurrence of negative life events in risk for suicidal ideation at age 17. Moreover, we documented how these effects vary according to biological sex.

Both univariable and multivariable results support the role of negative life events in suicidal ideation in boys and girls and the role of aggregate genetic liability specifically in girls. While genetic liability has been identified as an important factor in STB, only recently has genetic liability been supported at the genome‐wide level by well‐powered studies (Docherty et al., 2020; Mullins et al., 2021). The present research capitalizes on these recent advances to confirm the role of aggregate genetic liability for suicide attempts in adolescent girls, underscoring the importance of taking into account genetic liability and family history when evaluating STB risk. Differences in suicide‐related risk and predictors have been shown according to sex, with girls being at higher risk for suicide ideation and attempt (Miranda‐Mendizabal et al., 2019). This observation has been confirmed in adult twin studies evaluating the heritability of suicidal thoughts, attempt, and death across sex (Althoff et al., 2012; Edwards et al., 2021), but further exploration was needed using PGS among adolescents. Here, we found that suicidal thoughts are more likely to occur in girls with high genetic liability for suicide attempts. This observation provides molecular support for the association between suicidal ideation and behavior (Nock et al., 2009), though it is worth noting that STB may also have heterogeneous trajectories (Allan, Gros, Lancaster, Saulnier, & Stecker, 2019). Why the impact of genetic liability was limited to girls is unclear. While there is some evidence of higher heritability for suicide attempt among women (Edwards et al., 2021), we are unaware of a large‐scale genetic study of ideation with sex‐specific heritability estimates. Follow‐up studies are necessary to replicate this finding and determine whether this sex difference is limited to adolescence or persists into adulthood.

Regarding negative life events, our estimates highlight the strong role of bullying and drug use in suicidal ideation. The role of bullying is consistent with prior research investigating STB in adolescence (Holt et al., 2015; O'Reilly et al., 2021; Tang et al., 2020; Zaborskis et al., 2019). Bullying during adolescence can indeed be described as directly affecting the psychological foundation of the self, which is recognized as a major level of threat and constitutes a high‐risk factor for mental health issues (Brown & Harris, 1989). It is worth noting, however, that compared with other life events evaluated in this study, bullying is potentially a less discrete event, that is, it might continue across an extended time frame. Its potential duration in time may lead to prolonged distress (Shain, 2016), including STB that might be perceived as the only way to stop the threat. As for drug use, its strong positive association with adolescent suicidal ideation might be explained by a shared genetic liability with risk‐taking (Mullins et al., 2021), which has been corroborated in adolescence (Orri et al., 2020). Indeed, risk‐taking such as drug use or suicidal behavior may be used as ways to cope with emotional distress. The current result, showing that involvement with drugs in the past year increased suicidal ideation, is meaningful for risk screening and prevention. Drug use may indeed play a causal role by intensifying impulsive traits, which may be involved in the transition from suicidal thoughts to attempts (Mars et al., 2019b; O'Connor & Kirtley, 2018). We also observed that experiencing failures was associated with suicidal ideation. A previous review has discussed the association between suicide during adolescence and academic failure (Greydanus & Calles Jr., 2007), with failure being identified as a risk factor for suicide death. The current results support the role of school failures, especially in girls, and suggest that they can also be associated with risk of later suicidal ideation.

In addition to these common factors, we found specific associations between negative life events and suicidal ideation in boys and girls. First, both univariable and multivariable models underscored the strong role of recent parental death in boys. The association with parental death is in line with previous studies showing the adverse role of unfavorable emotional experiences during developmental periods (Burrell, Mehlum, & Qin, 2021; Hua et al., 2019), though this study expands on previous findings and supports the association between parental death during adolescence (i.e. death has been experienced between 16 and 17 years old) and STB. Surprisingly, this result was not observed in girls; that is, the association is not significant and the estimates are much lower than those observed in boys. This should, however, be interpreted with caution, as the number of parental deaths was low in our sample. Second, we observed that in the multivariable analysis, failure at school was associated with risk of suicidal ideation in girls but not in boys. This is in line with previous results showing that girls reported more suicidal ideation and anxiety at school than boys (Tan, Xia, & Reece, 2018), while we showed the role of this variable in the occurrence of suicidal ideation. Finally, whereas parental divorce has been associated with long‐term effects on mental health in a previous meta‐analysis (Auersperg, Vlasak, Ponocny, & Barth, 2019), we did not find evidence of this result. This might be related to the fact that we are evaluating divorce in late adolescence (Lindstrom & Rosvall, 2015) and need additional explorations.

As the association between suicidal ideation and aggregate genetic liability was observed only among girls, we limited our evaluation of potential interactions between PGS and the significant life events to that subsample. It is worth mentioning that these analyses did not reach the standard threshold used for significance in statistical analyses (*p* < .05) and warrant replications in more powerful samples. However, as the direction of these effects and their potential implications are informative, we have described interactions with *p* < .1. The current results suggested an interaction between PGS and drug use, indicating that the effect of past year drug use on suicidal ideation was exacerbated among girls with high genetic liability for suicide attempts. This is in line with previous studies showing the shared genetic liability between suicide attempts and drug use (Mullins et al., 2021) and the role of risk‐taking in suicidal behaviors (O'Connor & Kirtley, 2018; Orri et al., 2020). Unexpectedly, we also found indications that the risk of suicidal ideation in those who failed at school in the past year would be *lower* among girls with higher genetic liability to suicide attempts.

The present results should be interpreted in light of some limitations. First, the measure of suicidal ideation used in this study only covers the risk over the past week. Future studies should expand the measure of suicidal ideation over time and also include evaluations of suicide attempts. In addition, as we were particularly interested in the association and potential transition between suicidal thoughts and behaviors, our PGS measure was based on a GWAS for suicide attempts. Our results should thus be confirmed by using an evaluation of aggregate genetic risk specifically related to suicidal ideation when available, as these phenotypes present some specificities (Campos et al., 2020; Voracek & Loibl, 2007). Second, we have not performed corrections for multiple comparisons. One of the main reasons is that because of the correlations between our predictors, we cannot objectively determine how many independent tests were performed. Some of the observed effects might not survive corrections for multiple tests and thus warrant follow‐up in more powerful samples. Given the modest effect sizes we observed, especially for PGS, replication in independent samples is important to determine how to best incorporate these findings into prevention and intervention efforts. Nevertheless, it should be mentioned that this study presents a very conservative approach to offer a reliable understanding of the issue of STB in adolescence.

To conclude, this study evaluates the role of aggregate genetic liability and the occurrence of negative life events in the past year in association with suicidal ideation in adolescents. Our findings support the role of aggregate genetic liability in girls by using a PGS for suicide attempts. We also indicated the role of specific life events in suicidal ideation, with strong evidence for parental death in boys as well as bullying, drug use, and failure‐related events in both sexes. We found indications that genetic liability may interact with drug use and failure at school among girls, suggesting an interplay of genes and the environment in risk for suicidal ideation. These results offer perspectives for future research but also concrete avenues to target at‐risk adolescents in screening procedures, by paying attention to specific negative life events that may happen in adolescence and by considering the possible additional effects of genetic risk. While we did not use a PGS that specifically reflects suicidal ideation, our PGS has been computed based on a GWAS (Mullins et al., 2021) showing that the genetic etiology for suicide attempts is at least partially distinct from other psychiatric disorders. Given that suicide is a leading cause of death in youth (Shain, 2016), there is an urgent need to improve risk assessment and prevention efforts for STB. This could include a careful identification of potential bullying and the onset of drug use, especially in those with an elevated genetic propensity toward suicide attempts. Boys who were exposed to the death of a parent in the past year should also receive particular attention. Finally, we show the role of failure‐related events in both boys and girls, independently of genetic liability. Parents and other adults who interact with adolescents, including clinicians, should thus remain aware of negative emotions and possible suicidal ideation when adverse events happen at school or in an activity/group that is significant for the adolescent.

## Supporting information


**Figure S1.** Flowchart of participants' inclusion in the study. We focused on those who have been invited to the clinic assessment at 17 years old and first performed an Inverse Probability Weighting (IPW). Adolescents who were not included in the IPW computation did not have data on the predictors selected for this analysis (sex, SES, maternal psychopathology). After this procedure, we excluded participants with missing data for PGS (genetic data), suicidal ideation risk score, and negative life events. The final sample included 2,571 adolescents.
**Figure S2.** The interaction between aggregate genetic liability (PGS) and sex (boys on the left part and girls on the right part of the figure) in risk for suicidal ideation. Results indicate that girls with higher genetic liability reported higher suicidal ideation.
**Table S1.** Interaction between sex and PGS, and sex and negative life events in the prediction of suicidal ideation (*n* = 2,571).
**Table S2.** Univariable ordinal logistic regression models in boys (left; *n* = 1,135) and girls (right; *n* = 1,436) with suicidal ideation as outcome.
**Table S3.** Multivariable ordinal logistic regression models in boys (left; *n* = 1,135) and girls (right; *n* = 1,436) with suicidal ideation as outcome.
