# Supplementary material for: Suicidal ideation during adolescence: The roles of aggregate genetic liability for suicide attempts and negative life events in the past year
Source: J Child Psychol Psychiatry. 2022 Jun 29;63(10):1164–73. doi: 10.1111/jcpp.13653 (PMC9912194; doi:10.1111/jcpp.13653)
Supplement: Supplementary file 1 — Figure S1. Flowchart of participants' inclusion in the study. We focused on those who have been invited to the clinic assessment at 17 years old and first performed an Inverse Probability Weighting (IPW). Adolescents who were not included in the IPW computation did not have data on the predictors selected for this analysis (sex, SES, maternal psychopathology). After this procedure, we excluded participants with missing data for PGS (genetic data), suicidal ideation risk score, and negative life events. The final sample included 2,571 adolescents. Figure S2. The interaction between aggregate genetic liability (PGS) and sex (boys on the left part and girls on the right part of the figure) in risk for suicidal ideation. Results indicate that girls with higher genetic liability reported higher suicidal ideation. Table S1. Interaction between sex and PGS, and sex and negative life events in the prediction of suicidal ideation (n = 2,571). Table S2. Univariable ordinal logistic regression models in boys (left; n = 1,135) and girls (right; n = 1,436) with suicidal ideation as outcome. Table S3. Multivariable ordinal logistic regression models in boys (left; n = 1,135) and girls (right; n = 1,436) with suicidal ideation as outcome. [file JCPP-63-1164-s001.docx]

**SUPPORTING INFORMATION**

**Appendix S1. Methods**

***Participants***

In the current study, we focused on the clinical assessment youth completed at age 17 (Teen Focus 4). We included a subsample of individuals who have information on the continuous index for suicidal ideations, negative life events, and a PGS score (N = 2,571; see Figure S1). To correct for potential biases related to the selection of complete cases, we used Inverse Probability Weighting (Seaman & White, 2013) as a preliminary step in our analyses (IPW; see statistical analyses section for more details). In IPW, the analysis estimated on the sample with no missing data is weighted by the inverse of the probability of being a member of that sample, thus making the participants with no missing data representative of all individuals (Hughes, Heron, Sterne, & Tilling, 2019; Seaman & White, 2013).

***Polygenic liability***

Individuals were excluded on the basis of gender mismatches; minimal or excessive heterozygosity; disproportionate levels of individual missingness (>3%) and insufficient sample replication (IBD < 0.8). Population stratification was assessed by multidimensional scaling analysis and compared with Hapmap II (release 22) European descent (CEU), Han Chinese, Japanese and Yoruba reference populations; all individuals with non-European ancestry were removed. SNPs with a minor allele frequency of < 1%, a call rate of < 95% or evidence for violations of Hardy-Weinberg equilibrium (P < 5E-7) were removed. Cryptic relatedness was measured as proportion of identity by descent (IBD > 0.1). Related subjects that passed all other quality control thresholds were retained during subsequent phasing and imputation. 9,115 subjects and 500,527 SNPs passed these quality control filters. Genotype data were imputed to the Haplotype Reference Consortium (HRC) reference panel16 (McCarthy et al., 2016).

***Statistical analysis***

To control for potential biases related to the deletion of missing data, we used an IPW (Seaman & White, 2011) with clinic attendance as a binary outcome. Predictors of clinic attendance were selected among the earliest waves of data collection to minimize missingness. Predictors included participants’ sex, SES (housing tenure and maternal education level), and maternal psychopathology. Housing tenure evaluated the residential situation of the family with a score going from 0 (owned a home, with a mortgage) to 5 (rented a home from a housing association). Only a small amount of missing data has been found for this variable and were thus recoded to the modal value, following previous recommendations (Seaman & White, 2011). Maternal psychopathology was evaluated with the Edinburgh Post-Natal Depression Scale (EPDS) (Cox, Holden, & Sagovsky, 1987). EPDS scores included in this analysis have been combined from two waves of data collection (at 18 weeks of gestation and when the child was 8 weeks old). The IPW was computed with the “ipwpoint” function of the R package IPW (<https://cran.r-project.org/web/packages/ipw/index.html>). Individual weights were retrieved from this analysis and used in subsequent regression models to make the complete cases representative of all cases.

**Appendix S2. Results**

Sensitivity analysis conducted using univariable and multivariable ordinal logistic models showed similar results than the ones described in the main paper, conducted with gaussian models (see Tables S2 and S3).

**
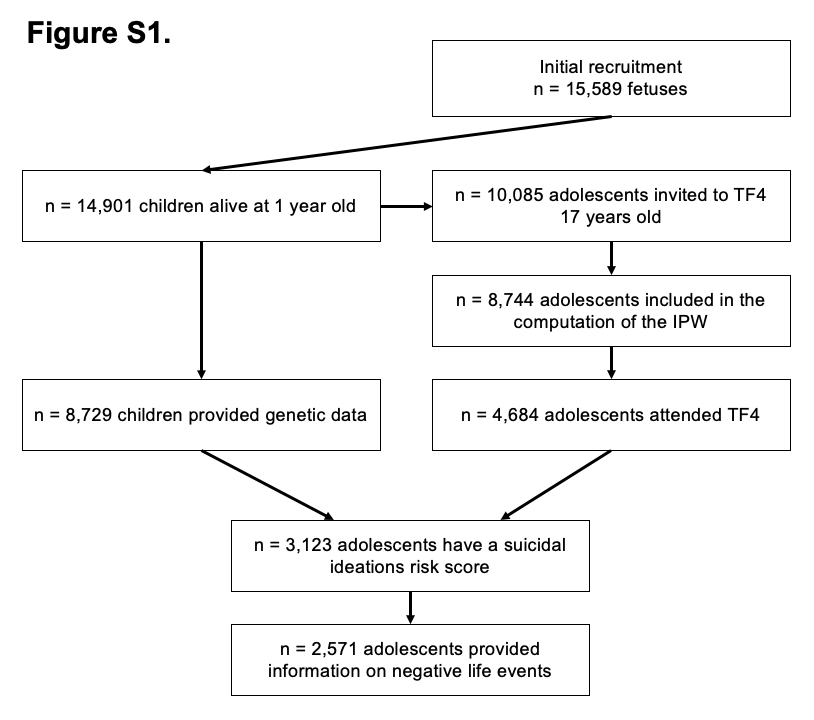
**

***Note***. Figure S1 is a flowchart of participants’ inclusion in the study. We focused on those who have been invited to the clinic assessment at 17 years old and first performed an Inverse Probability Weighting (IPW). Adolescents who were not included in the IPW computation did not have data on the predictors selected for this analysis (sex, SES, maternal psychopathology). After this procedure, we excluded participants with missing data for PGS (genetic data), suicidal ideation risk score, and negative life events. The final sample included 2,571 adolescents.

**Table S1.** Interaction between sex and PGS, and sex and negative life events in the prediction of suicidal ideation (n=2,571)

|  | **Estimate** | ***t*** | ***p*-value** |
| --- | --- | --- | --- |
| Sex | 0.217 | 7.67 | <0.001 |
| PGS | -0.005 | 0.26 | 0.792 |
| Sex*PGS | 0.062 | 2.22 | 0.026 |
|  |  |  |  |
| Sex | 0.228 | 7.72 | <0.001 |
| Drug use | 0.384 | 6.62 | <0.001 |
| Sex*Drug | 0.089 | 0.97 | 0.330 |
|  |  |  |  |
| Sex | 0.201 | 7.01 | <0.001 |
| Bullying | 0.362 | 3.73 | <0.001 |
| Sex*Bullying | 0.266 | 2.00 | 0.045 |
|  |  |  |  |
| Sex | 0.219 | 7.73 | <0.001 |
| Parental death | 1.023 | 2.50 | 0.012 |
| Sex*Parental death | -0.998 | 1.88 | 0.061 |
|  |  |  |  |
| Sex | 0.216 | 7.10 | <0.001 |
| Grand-parent death | 0.037 | 0.64 | 0.525 |
| Sex* Grand-parent death | 0.009 | 0.11 | 0.910 |
|  |  |  |  |
| Sex | 0.209 | 7.13 | <0.001 |
| Friend death | 0.079 | 0.88 | 0.379 |
| Sex*Friend death | 0.086 | 0.73 | 0.468 |
|  |  |  |  |
| Sex | 0.220 | 7.33 | <0.001 |
| Parental hospitalization | 0.132 | 2.13 | 0.033 |
| Sex*Parental hospitalization | -0.010 | 0.11 | 0.913 |
|  |  |  |  |
| Sex | 0.220 | 7.66 | <0.001 |
| Parental divorce | 0.110 | 0.79 | 0.428 |
| Sex*Parental divorce | -0.133 | 0.66 | 0.509 |
|  |  |  |  |
| Sex | 0.212 | 7.16 | <0.001 |
| Friend injury | 0.144 | 1.83 | 0.068 |
| Sex*Friend injury | 0.007 | 0.07 | 0.945 |
|  |  |  |  |
| Sex | 0.190 | 5.76 | <0.001 |
| Failure achievement | 0.259 | 5.91 | <0.001 |
| Sex*Failure achievement | 0.084 | 1.37 | 0.172 |
|  |  |  |  |
| Sex | 0.202 | 6.57 | <0.001 |
| Failure at school | 0.129 | 2.33 | 0.020 |
| Sex*Failure at school | 0.103 | 1.31 | 0.189 |


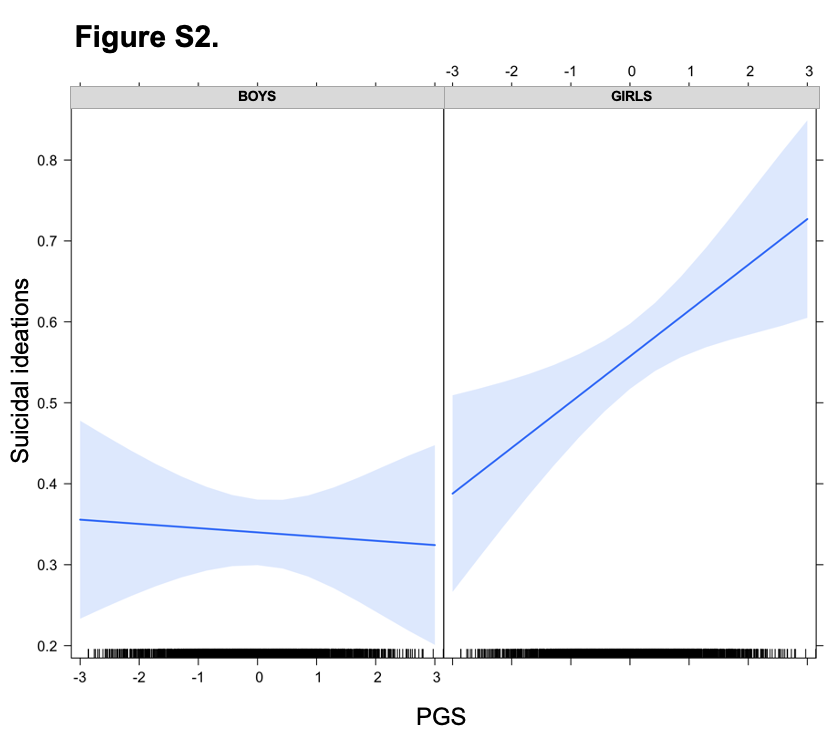


***Note***. Figure S2 shows the interaction between aggregate genetic liability (PGS) and sex (boys on the left part and girls on the right part of the figure) in risk for suicidal ideation. Results indicate that girls with higher genetic liability reported higher suicidal ideation.

**Table S2.** Univariable ordinal logistic regression models in boys (left; n=1,135) and girls (right; n=1,436) with suicidal ideation as outcome

|  | ***BOYS*** | | | ***GIRLS*** | | |
| --- | --- | --- | --- | --- | --- | --- |
|  | ***Estimate*** | ***z*** | ***p-value*** | ***Estimate*** | ***z*** | ***p-value*** |
| SES | 0.051 | 2.25 | 0.024 | -0.008 | 0.16 | 0.873 |
| PGS | 0.047 | 0.73 | 0.464 | 0.135 | 2.48 | 0.013 |
| Drug use | 1.006 | 5.83 | <0.001 | 1.001 | 5.18 | <0.001 |
| Bullying | 0.977 | 3.60 | <0.001 | 1.429 | 6.04 | <0.001 |
| Parental death | 2.334 | 2.55 | 0.011 | 0.320 | 0.38 | 0.702 |
| Grand-parent death | 0.101 | 0.54 | 0.587 | 0.104 | 0.62 | 0.532 |
| Friend death | 0.237 | 0.85 | 0.397 | 0.354 | 1.69 | 0.091 |
| Parental hospitalization | 0.416 | 2.23 | 0.026 | 0.264 | 1.46 | 0.144 |
| Parental divorce | 0.349 | 0.83 | 0.407 | -0.209 | 0.48 | 0.632 |
| Friend injury | 0.596 | 2.66 | 0.008 | 0.366 | 2.03 | 0.042 |
| Failure achievement | 0.878 | 6.43 | <0.001 | 0.883 | 7.34 | <0.001 |
| Failure at school | 0.514 | 3.12 | 0.002 | 0.581 | 3.90 | <0.001 |

*Note*. The left part of the Table shows univariable results in boys and the right part of the Table indicates univariable results in girls.

**Table S3.** Multivariable ordinal logistic regression models in boys (left; n=1,135) and girls (right; n=1,436) with suicidal ideation as outcome

|  | ***BOYS*** | | | ***GIRLS*** | | |
| --- | --- | --- | --- | --- | --- | --- |
|  | **Estimate** | ***z*** | ***p*-value** | **Estimate** | ***z*** | ***p*-value** |
| SES | 0.038 | 1.67 | 0.094 | -0.007 | 0.13 | 0.896 |
| PGS | 0.046 | 0.70 | 0.487 | 0.110 | 1.97 | 0.048 |
| Drug use | 0.843 | 4.77 | <0.001 | 0.883 | 4.45 | <0.001 |
| Bullying | 0.834 | 3.03 | 0.002 | 1.071 | 4.35 | <0.001 |
| Parental death | 2.188 | 2.35 | 0.019 | 0.438 | 0.52 | 0.606 |
| Grand-parent death | 0.129 | 0.68 | 0.499 | 0.063 | 0.37 | 0.712 |
| Friend death | -0.026 | 0.09 | 0.931 | 0.221 | 0.99 | 0.324 |
| Parental hospitalization | 0.337 | 1.75 | 0.080 | 0.293 | 1.55 | 0.122 |
| Parental divorce | 0.191 | 0.44 | 0.660 | -0.169 | 0.37 | 0.709 |
| Friend injury | 0.442 | 1.90 | 0.056 | 0.012 | 0.06 | 0.952 |
| Failure achievement | 0.705 | 4.91 | <0.001 | 0.766 | 6.08 | <0.001 |
| Failure at school | 0.306 | 1.79 | 0.079 | 0.310 | 1.97 | 0.048 |

*Note*. The left part of the Table shows multivariable results in boys and the right part of the Table indicates multivariable results in girls

**Supplementary references**

Cox, J. L., Holden, J. M., & Sagovsky, R. (1987). Detection of Postnatal Depression. Development of the 10-item Edinburgh Postnatal Depression Scale. *British Journal of Psychiatry, 150*, 782-786. doi:<https://doi:10.1192/bjp.150.6.782>

McCarthy, S., Das, S., Kretzschmar, W., Delaneau, O., Wood, A. R., Teumer, A., . . . Haplotype Reference, C. (2016). A reference panel of 64,976 haplotypes for genotype imputation. *Nature Genetics, 48*(10), 1279-1283. doi:10.1038/ng.3643

Seaman, S. R., & White, I. R. (2011). Review of inverse probability weighting for dealing with missing data. *Stat Methods Med Res, 22*(3), 278-295. doi:10.1177/0962280210395740
